# Scleredema Diabeticorum in a Patient with Type 2 Diabetes Mellitus

**DOI:** 10.1155/2011/560273

**Published:** 2011-07-31

**Authors:** C. Martín, L. Requena, K. Manrique, F. D. Manzarbeitia, A. Rovira

**Affiliations:** ^1^Endocrinology and Nutrition Department, Fundación Jiménez Díaz-Capio, 28040 Madrid, Spain; ^2^Dermatology Department, Fundación Jiménez Díaz-Capio, 28040 Madrid, Spain; ^3^Pathological Anatomy Department, Fundación Jiménez Díaz-Capio, 28040 Madrid, Spain

## Abstract

*Background. *Scleredema adultorum, a connective tissue disorder of unknown aetiology, is characterized by a thickening of the reticular dermis in the upper back of the body that may decrease the mobility of the affected tissues. It has been reported in diabetic patients with poor metabolic control. Therapeutic options are limited with generally poor results. *Case Report. *53-year-old white male with type 2 diabetes mellitus was referred to our department for evaluation of incipient nephropathy and retinopathy. On examination, he presented erythematous, indurated, painless and ill-defined plaque on the skin of the upper back with limited movement of shoulders. A biopsy was done revealing scleredema. PUVA treatment and physiotherapy were started with the amelioration of mobility and acquiring some elasticity of the upper back. *Discussion. *The development of scleredema in diabetic patients has been related to prolonged exposure to chronic hyperglycaemia. Our patient has had diabetes for 20 years with an acceptable glucose control, however he developed the scleredema 10 years ago. *Conclusions. *Scleredema is a rare connective disorder that seems to appear most frequently in diabetic subjects. Good metabolic control seems not to preclude its development. PUVA treatment and physiotherapy are therapeutic options that seem to be of some help.

## 1. Introduction

Scleredema diabeticorum is an infrequent connective tissue disorder described for the first time by Buschke in the year 1900. It develops in the skin on the upper part of the back, shoulders, and neck. In rare occasions, the disease involves the face, arms, and the rest of the trunk, occasionally the viscera may be affected, but not the hands and the feet. Scleredema diabeticorum is characterized by thickening, hardening, and painlessness of the affected skin. There is no clear demarcation between involved and normal skin. Sometimes, it has been associated with erythema and pigmentation of the skin. Severe cases of scleredema may present a restrictive defect in pulmonary function [1].

There have been described three variants of scleredema [1]. Type 1 develops after an acute febrile illness, mostly of streptococcus origin. This type of scleredema usually disappears over several months. Type 2 scleredema appears in subjects without diabetes or infection. Type 3 scleredema develops in subjects with type 1 or type 2 diabetes mellitus of long duration, treated with insulin, with poor metabolic control, with obesity, and with classic complications of diabetes. This third type is called scleredema diabeticorum or scleredema adultorum of Buschke [2]. 

The pathogenesis of the scleredema diabeticorum remains unclear. It has been proposed that nonenzymatic glycosylation of collagen fibers may alter its degradation. Other hypothesis suggests that glucose may stimulate fibroblast proliferation and the synthesis of extracellular matrix components. Immunological response has also been postulated, as some patients have ameliorated following treatment with cyclosporine, but the lack of lymphocytic infiltrates in the dermal lesions seems to rule out a T-cell-mediated etiologic mechanism.

Several treatments have been used although the disease is refractory to them in most of the cases. The therapies used include antibiotic, corticosteroids, chemotherapy, radiation, tight glycemic control, PUVA, and UV-A1 therapy. The best result has been obtained with the last two therapies. We here report scleredema in a patient with type 2 diabetes mellitus and good metabolic control recently observed in our hospital.

## 2. Case Report

**Figure 1:**
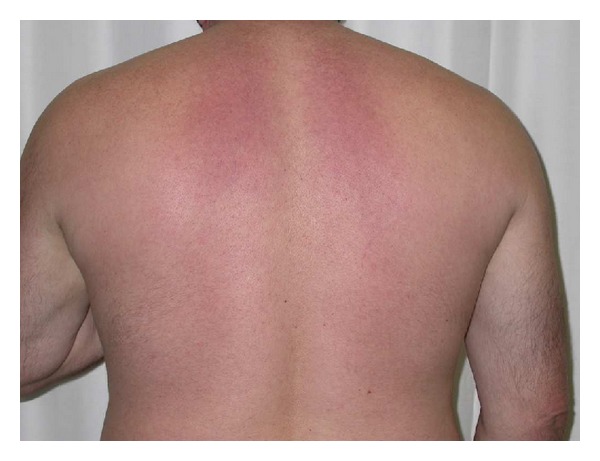
Thickening and erythema of the upper part of the back.

A 53-year-old white man, suffered from type 2 diabetes diagnosed 20 years ago, was referred to our department for evaluation of recent discovery of incipient nephropathy and retinopathy. He was previously visiting another endocrinologist in the town but because of a change of job, he moved into the city. He brought a letter specifying that his glucose control had been acceptable since the diagnosis of diabetes (HbA1c lower than 7%). Recently, he was diagnosed of incipient nephropathy (microalbuminuria 200 mg/24 hours), high systolic blood pressure (140 mmHg), high LDL cholesterol (134 mg/dL), and retinal hard exudates close to the macula treated with laser photocoagulation. His current medication included metformin (2.550 mg/day), Detemir insulin (50 U/day), premeals Aspart insulin, simvastatin (20 mg/day), Candesartan (16 mg/day), and AAS (100 mg/d). Physical examination revealed the following: weight of 120 Kg, height of 174 cm, body index mass of 30,27 Kg/m^2^, blood pressure of 150/75 mmHg, and normal auscultation. The skin of the upper back and posterior neck was erythematous, indurated, and painless (Figure 1) with moderate restriction of range of motion of the shoulders and neck. 

Blood analysis revealed the following: leukocytes of 7500 *μ*L, haemoglobin of 16,3 g/dL, platelets of 281000  *μ*L, sedimentation glomerular rate of 10 mm, glucose of 135 mg/dL, creatinine of 0,3 mg/dL, cholesterol of 157 mg/dL, HDL-c of 58 mg/dL, LDL-c of 81 mg/dL, triglycerydes of 90 mg/dL, C peptide of 3,8 ng/mL, TSH of 1,78 *μ*UI/mL, negative antithyroid antibodies, microalbuminuria of 240 mg/24 h, and HbA1c of 6,7%. The serum protein electrophoresis was normal, excluding monoclonal gammopathy.

**Figure 2:**
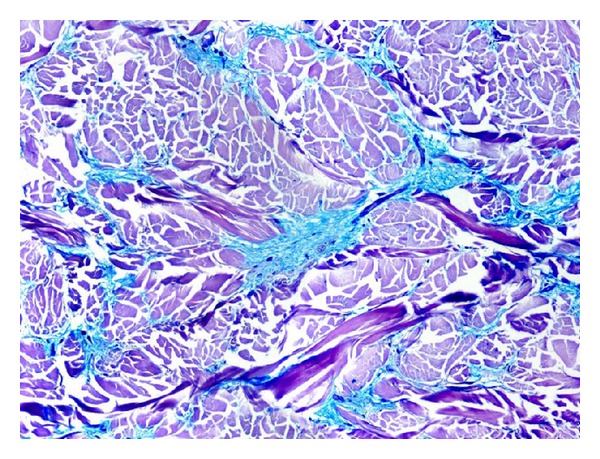
Histological aspect of skin biopsy. Blue Alcian dye. Thickened dermis and increased accumulation of aminoglycans between large collagen bundles.

When we noticed the lesion on the skin and asked about it, the patient explained that for the last 10 years he had noticed a progressive hardening of the skin of this area, that became less sensitive, and he also noticed a decrease in motility of his neck and shoulders. He did not remember if he was febrile 10 years ago when the lesion appeared on his back. He had never been studied for the skin disorder that he related with his obesity. The patient was sent to the dermatologist who suspected the diagnosis of scleredema and performed a skin biopsy. Histopathologic study demonstrated thick collagen bundles separated by spaces filled with mucin in the deep reticular dermis, consistent with scleredema adultorum of Buschke (Figure 2). He recommended physiotherapy and UV-A1 therapy. The latter treatment was not made because of a lack of this modality of phototherapy in his town, then he started PUVA therapy. 

After two months of PUVA therapy (total cumulative UVA dose 120 J/cm^2^) and physical exercises, the patient has noticed amelioration of the mobility of the back and shoulders, and on exploration, the redness of the upper back has disappeared, and the skin was softer.

## 3. Discussion

The series of clinical cases of scleredema in diabetic patients published until now include subjects with the following characteristics: long duration of diabetes, poor glucose control, diabetic microangiopathy, obesity, and insulin treatment [1, 3]. Our patient presented all of these factors except for a reasonable good glucose control since the beginning of diabetes. Although the cause of scleredema remains unknown, the most consistent hypothesis is that chronic hyperglycaemia progressively damages the collagen fibres of the connective tissue, by a nonenzymatic glycosylation process, accumulating in the dermis and then causing the scleredema. However, this mechanism does not seem to be, at least solely, the cause of scleredema in the case that we present here. The incipient signs of microvascular complications of diabetes, such as microalbuminuria and hard exudates in the retina, appeared later on the course of the development of the skin disorder. Therefore, other mechanisms should also account for causing the lesion; otherwise, a more susceptible ground for developing such complications may exist in some patients. 

The diagnosis of scleredema is generally suspected on a clinical base; however, the definitive diagnosis is obtained by skin biopsy. Microscopic features of the biopsy are characterized by thickening of the dermis due to enlarged collagen bundles in deep reticular dermis with clear spaces between them, filled with mucin. However, mucin deposits are inconstant and not necessary for the diagnosis [4].

It has been reported that monoclonal gammopathy has developed in some patients with scleredema, specially, without diabetes, even many years after the appearance of the skin lesion [5]. It is recommendable to perform a serum protein electrophoresis with an annual periodicity in those patients.

Different treatment modalities for scleredema have been reported as case reports or small series with variable success. When infection is confirmed (type 1 or 2 scleredema), antibiotics can be used, but they are unnecessary in type 3 scleredema. In most cases of this specific scleredema diabeticorum, glucose control intensification has been the first step in the treatment. In one series of four type 1 diabetics, a decrease of HbA1c from 9.3 to 7.9% produced an amelioration of the scleredema [6]. In another series of diabetic patients, in five out of eleven patients, the scleredema lesions improved partially with good glucose control [3]. Other reports did not found a better course of the skin lesion after improving glucose control [7, 8]. Immunosuppressive therapy has also been assayed in some patients with scleredema. Cyclosporine, corticoids, and methotrexate have produced inconsistent results [9–12]. Radiation therapy is another treatment modality. Severe restrictive scleredema associated to type 2 diabetes has been shown to improve after electron-beam radiotherapy although the effect was not consistently durable [13]. Most recently, ultraviolet A-1 phototherapy has become available for a variety of skin diseases. The first case of scleredema diabeticorum successfully treated with UVA-1 was published in 2004 [14]. Since then, six more patients have been reported of being treated with UVA-1, only one dropped out due to a polymorphic light eruption reaction, and the remainder presented good responses [15]. We recommended UVA-1 therapy to our patient, but it was not available in his town, therefore he started with oral photochemotherapy using oral psoralen plus ultraviolet A (PUVA) therapy. This type of treatment has been reported to be effective in patients with scleredema adultorum, as it seems to work in our patient [16]. The mechanism of the benefit of PUVA on scleredema may be related to an increase in collagenase synthesis by fibroblasts and by inhibiting de novo synthesis of type I collagen [17]. In a review by Brenner et al. [18], they concluded that because of the paucity of valid therapeutic alternatives, phototherapy and photochemotherapy with UVA1 or PUVA may also be warranted and useful in several sclerosing skin diseases like genital lichen sclerosus and atrophicus, scleredema adultorum of Buschke, scleromyxedema, or necrobiosis lipoidica.

## 4. Conclusion

Scleredema adultorum is a rare disorder that may develop in diabetic patients with poor metabolic control. However, it may also occur in diabetic subjects with quite good glucose control as it happened in the present case. Other etiologic mechanism besides hyperglycemia probably takes place in some patients. There are different approaches for the treatment of scleredema adultorum. Tight glycemic control is recommended but has not proven effective on skin lesions once they exist. Ultraviolet A-1 phototherapy and photochemotherapy with PUVA seem to be the most effective treatments for this pathology.

## Abbreviations

HbA1c: Glycated haemoglobin
UVA-1: Ultraviolet A-1 phototherapy
PUVA: Psoralen plus ultraviolet photochemotherapy
AAS: Acetylsalicylic acid.
